# Correlation between Testicular Hemodynamic and Semen Quality Indices in Clinical Varicocele Patients in Pakistan

**DOI:** 10.1155/2019/7934328

**Published:** 2019-03-07

**Authors:** Khaleeq Ur Rehman, Hafsa Zaneb, Abdul Basit Qureshi, Muhammad Shahbaz Yousaf, Ahsan Numan, Khalid Abdul Majeed, Imtiaz Rabbani, Tahir Mehmood Khan, Habib Rehman

**Affiliations:** ^1^Department of Urology and Andrology, Fatima Memorial College of Medicine and Dentistry, Lahore, Pakistan; ^2^Department of Physiology, University of Veterinary and Animal Sciences, Lahore, Pakistan; ^3^Department of Anatomy and Histology, University of Veterinary and Animal Sciences, Lahore, Pakistan; ^4^Department of Surgery, Services Institute of Medical Sciences, Lahore, Pakistan; ^5^Department of Neurology, Services Institute of Medical Sciences, Lahore, Pakistan; ^6^Institute of Pharmaceutical Sciences, University of Veterinary and Animal Sciences, Lahore, Pakistan

## Abstract

Varicocele, a vascular event, is associated with infertility due to testicular damage that causes abnormal spermatogenesis in males. The goal of this study is to ascertain the diagnostic significance of scrotal color Doppler ultrasonography (CDUS) by measuring peak systolic value (PSV) and resistive index (RI) of the arteries supplying blood to the testis and their association with semen quality attributes. Sixty prospective patients (age: 20-50 years) undergoing microsurgical varicocelectomy at a teaching hospital were included in the study. Semen parameters and CDUS were recorded and testicular blood flow was determined as PSV and RI of subcapsular artery and intraparenchymal artery of the testes. Nonparametric statistics was applied to test the correlation/association of the semen quality with the PSV, RI, and other variables. Results revealed a significant negative correlation (r = -0.28; p < 0.05) between progressive motility of spermatozoa and resistive index of the intraparenchymal arterial blood flow. Furthermore, it was noticed that the progressive motility of spermatozoa was tended to be negatively correlated (r = -0.236) with resistive index of subcapsular arterial blood flow. In conclusion, this study has revealed that progressive motility of sperms has correlation with the intraparenchymal blood flow of testes. The progressive motility of sperms could be correlated with RI of testicular blood flow. The apparent lack of association between diameter of varicocele vein and semen quality signifies the need of investigating some other factors that may be involved in pathogenicity of varicocele. The diagnostic value of CDUS may be carefully interpreted and clinically correlated in assessment of severity of varicocele.

## 1. Introduction

Varicocele, a common cause of infertility in males, affects 15% male population in general and 21-39% in subfertile men [1–3]. Although its pathophysiology is not clear, it is characterized by progressive testicular damage, reduced testicular volume, and Leydig cell dysfunction [4]. It is becuase poor venous hemodynamics causes retrograde flow in testicular veins which interferes with thermoregulatory mechanisms in spermatic cord. This can lead to various anomalies associated with varicocele like decreased testosterone synthesis and spermatogenesis. An understanding of the dynamics by which varicocele can influence an individual can assist in devising efficient treatment strategy [5]. It has been found that semen quality parameters were negatively associated with grade of varicocele [6] and were improved after successful microsurgical varicocelectomy [7].

Most of the research, in the last few decades, to understand the pathophysiology of varicocele has been focused on vascular structures. Color Doppler ultrasonography (CDUS) is the standard diagnostic tool in assessment of varicocele [8]. Generally, the diagnosis of varicocele is made clinically by physical examination, having only specificity of 70% and confirmed by CDUS analysis (sensitivity 97% and specificity 94%). This radiographic tool also assesses the severity of the varicocele by characterizing the venous diameter, presence/absence of blood reflux, and other associated parameters. Several studies have deduced correlation of ultrasound findings and varicocele [9–11], but the value of ultrasonography for evaluation of varicocele is still controversial [12, 13] and, therefore, needs further research.

We have hypothesized that abnormal testicular blood flow on CDUS in varicocele patient will have impaired semen quality. Therefore, we investigated the hemodynamic parameters of testicular subcapsular and intratesticular arteries from clinically diagnosed varicocele patients, such as resistivity index (RI) and peak systolic velocity (PSV), and correlated them with selected semen quality parameters.

## 2. Materials and Methods

The study was approved by the Advanced Studies and Research Board, University of Veterinary and Animal Science, Lahore, and Institutional Review Board of Fatima Memorial College of Medicine and Dentistry, Lahore, Pakistan.

### 2.1. Subject Selection

Sixty consecutive patients, diagnosed with infertility and varicocele, undergoing microsurgical varicocelectomy were included in the study after written informed consent.

### 2.2. Inclusion Criteria

Individuals with age 20-50 years, with grade two or three varicocele on physical examination [14] by a urologist, and with varicocele vein diameter of >2.5mm on scrotal CDUS [15] were included in the study.

### 2.3. Exclusion Criteria

Patients with the following comorbid conditions were excluded from the study: any chronic illness (Hepatitis C or B patients, patients on prolonged drugs that have side effects for spermatogenesis, e.g., antiviral drugs, chemotherapeutic agents), patients with hormonal disorders (hypogonadism, hypothyroidism, etc.), patients on prolonged antidepressants or drugs addicts, patients with male or female sexual dysfunction leading to decreased frequency of intercourse to less than twice per week, patients with a significant female factor infertility that could be held responsible for the couple's infertility.

### 2.4. Semen Analysis

Semen samples from the individuals were collected at three to four days of sexual abstinence and were processed for the determination of semen quality parameters including sperm count, motility, and normal morphology according to standard reference limits for semen characteristics [14].

### 2.5. Scrotal Ultrasonography

All the scrotal CDUS examinations were performed by the same radiologist while using a color Doppler ultrasonography machine (Voluson General Electronics 30, USA) with 7.5-10 MHz linear probe [14, 16]. Briefly, grey scale ultrasonography was done to detect any other associated abnormalities. Testicular echogenicity and homogeneity were assessed in grey scale US in supine positions. Testicular diameter (both longitudinal and transverse diameters) and transverse epididymal diameter were measured. Blood flow of the subcapsular branch of testicular artery and testicular intraparenchymal were recorded. Peak systolic velocities (PSV) and resistive indices (RI) were determined for both arteries. The RI was calculated by the formula (peak systolic velocity – end diastolic velocity)/ peak systolic velocity. Varicocele vein diameter and the backflow status of varicocele veins were noted both in lying and standing position. Three separate measurements for each of the variable studied were made and subsequently averaged. The grading and the interpretation of the results were performed as described earlier [16, 17].

### 2.6. Statistical Analysis

Data were presented as means ± standard deviation. The normal distribution of data was evaluated by Shapiro-Wilk test. The Pearson's Correlation test was used to determine correlations between the variables with the help of statistical package for social sciences (SPSS version 20) with significance predetermined at < 0.05, while p < 0.1 was considered as tendency.

## 3. Results

**Figure 1:**
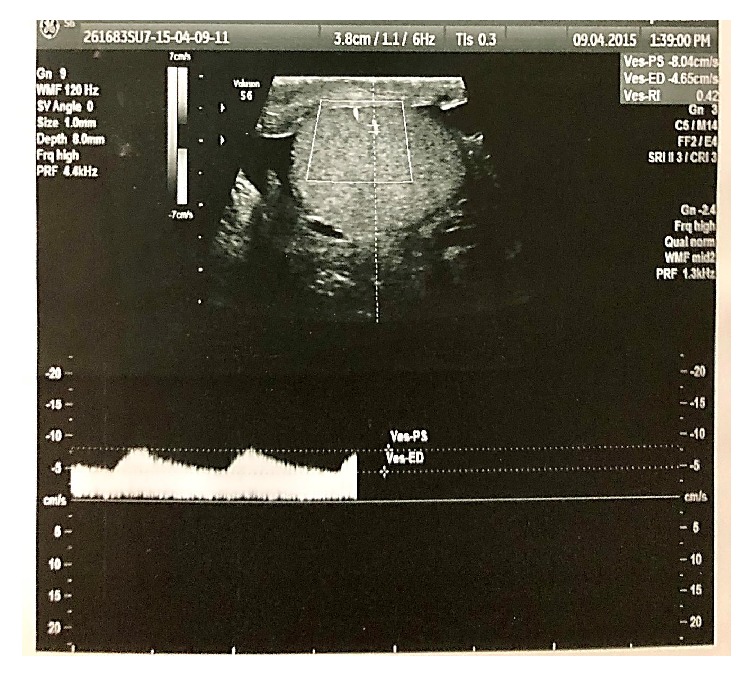
Doppler ultrasonographic measurement of the peak systolic velocity (PSV) and resistive index (RI) of the intraparenchymal testicular artery.

Mean age of the patients involved in the study was 31.77 ± 7.48, ranging from 20 to 50 years. The sperm count, progressive motility, nonprogressive motility, and immotile and normal morphology of spermatozoa of the patients were 32.42 ± 32.51 million/mL, 16.47 ± 14.53%, 18.70 ± 13.22%, 60.37 ± 25.24%, and 5.18 ± 5.47%, respectively (Table 1). The variables of left testes, determined by CDUS, are summarized in Table 2. No significant correlation was observed between diameters of varicocele veins (Figure 1) both at lying and at standing positions with parameters of semen quality (Table 3). However, progressive motility tended to exhibit a weak correlation (p = 0.079, r = 0.228) with diameter of varicocele vein at standing position. Indices of blood flow pattern also showed nonsignificant correlations with various determinants of semen quality. A significant negative correlation (r = -0.28; p < 0.05) between progressive sperm motility and resistive index of the testicular intraparenchymal artery (Table 4) was observed in varicocele patients. Semen quality parameters also showed nonsignificant correlations with size of testes (both longitudinal and transverse diameters) and transverse epidydimal diameter (Table 5).

## 4. Discussion

It has been reported that there is a general relationship between ultrasound findings and semen parameters in varicocele patients [9] and it is used for the diagnosis of infertile patients [18, 19] although further standardization is yet required [20]. The CDUS has 83% to 95% sensitivity for identifying subclinical varicocele. Clinical varicocele diameter of > 2.45 mm at rest and >2.95 mm during valsalva maneuver has a high predictive value for having a clinical varicocele [21]. In the present study, we included those infertile patients having >2.5 mm varicocele vein diameter on CDUS to avoid any bias. Moreover, all the patients were diagnosed as infertile based on having one or more abnormalities of semen. It is suggested that venous stasis that leads to increased intrascrotal pressure is an important etiology contributing to infertility in clinical varicocele patients. This decreased venous stasis leads to compromised thermodynamics and loss of cooling effect to maintain low intrascrotal temperature in outgoing venous plexus. However, the exact mechanism of how the temperature affects spermatogenesis remains unclear. Although testicular venous pH, oxygen, and carbon dioxide pressure measurements were found to be insufficient to show venous stasis effect, venous hypertension in peritesticular veins was reported as the constant feature of varicocele. Presumably, this hypertension is responsible for the pathological changes in cord vein and testis [22].

The testes receive arterial blood supply via testicular, vasal, and cremasteric arteries. These arteries anastomose before entering into the testes so that sufficient amount of blood may be supplied to the organ. CDUS is a noninvasive tool for investigating the vascular structures and the associated pathologies with high sensitivity [8]. It was presumed that these varicocele patients will have correlation between testicular blood flow and semen quality parameters. Therefore, we attempted to explore the relationship between blood flow pattern and semen quality disorders. Our study revealed negative correlations only between progressive motility and RI of subcapsular (r = -0.236; p = 0.07) and intraparenchymal arteries (r = -0.28; p = 0.02). Our results are partially in agreement with the findings of Semiz et al. (2014) who failed to demonstrate any relationship between hemodynamic pattern of blood flow and semen analysis parameters, except a significant relationship between PSV in testicular arteries and semen count in patients with clinical varicocele [10]. This apparent discrepancy in the results may be attributed to the site of CDUS measurement. Therefore, it appears that Doppler indices are not predictive parameters to assess the extent of clinical varicocele cases in our study circumstances. Recently, we found that increased intravascular pressure of internal spermatic varicocele veins has negative correlation with sperm motility, morphology, and testicular blood flow [23].

Similarly, there was no significant relationship between testicular size and sperm disorders in the current study. Mehdaviet et al. (2016) also recoded similar findings in terms of nonsignificant correlation between testicular size and semen analysis parameters in patients with varicocele. It has been reported that change in diameter of varicocele veins between lying and standing position has high diagnostic accuracy to predict abnormalities of semen quality [9]. Therefore, we also presumed that larger varicoceles would have greater disorders of spermatogenesis. We could not confirm this finding in our patients except a tendency pattern (p = 0.07) weak association (r = 0.228) between progressive motility and varicocele diameter in standing position. Contrary to our findings, significant correlation between varicocele vein diameter and sperm count motility and morphology has been observed [11]. According to our results, the extent of damage to spermatozoa cannot be predicted by the diameter of varicocele veins.

## 5. Conclusion

We have found that the progressive motility of sperms has correlation with the resistive index of testicular blood flow although sperm count has no correlation with testicular blood flow. The apparent lack of association between varicocele vein diameter and semen quality shows that some other factors are also involved in the pathogenicity of the varicocele that needed to be investigated. Moreover, the diagnostic value of CDUS may carefully be interpreted and may be clinically correlated before diagnosing the severity of varicocele.

## Figures and Tables

**Table 1:** Semen analysis parameters of varicocele patients (n = 60).

Parameter	Mean ± SD	Minimum-Maximum
Count (million/mL)	32.42 ± 32.51	00.00-199.09
Progressive motility (%)	16.47 ± 14.53	00.00-65.00
Non progressive motility (%)	18.70 ± 13.22	00.00-50.00
Immotile (%)	60.37 ± 25.24	00.01-100.00
Morphology (%)	5.18 ± 5.47	00.00-25.00

**Table 2:** Spectral Doppler ultrasonographic recordings of left testis (n = 60).

Parameter	Mean ± SD	Minimum-Maximum
Longitudinal Diameter (mm)	40.43 ± 4.20	29.00-50.00
Transverse Diameter (mm)	19.90 ± 2.84	14.00-27.00
Epididymal Diameter (mm)	7.14 ± 1.54	3.50-11.50
Varicocele Lying Diameter (mm)	3.26 ± 0.85	2.20-5.50
Varicocele Standing Diameter (mm)	3.72 ± 1.10	2.50-7.70
PSV of Subcapsular artery (cm/sec)	6.31 ± 1.32	4.22-9.50
PSV of Intraparenchymal artery (cm/sec)	5.44 ±1.74	0.00-11.71
RI of Subcapsular artery	0.54 ± 0.10	0.32-00.74
RI of Intracapsular artery	0.50 ± 0.11	0.26-1.00

PSV: peak systolic velocity; RI: resistive index.

**Table 3:** Correlation coefficient (r) between semen quality parameters and diameters of varicocele veins in varicocele patients (n = 60).

Parameter		Varicocele Vein Diameter	
		Laying Position	Standing Position
Sperm count	r	-0.031	-0.058
	p	0.815	0.663
Progressive motility	r	0.181	0.228
	p	0.166	0.079
Non-progressive motility	r	-0.004	0.044
	p	0.978	0.739
Immotile	r	-0.127	-0.111
	p	0.335	0.401
Morphology	r	0.017	-0.038
	p	0.899	0.776

p stands for probability value.

**Table 4:** Correlation coefficient (r) between semen quality parameters with indices of testicular blood flow pattern in varicocele patients (n = 60).

Parameter		Sub-capsular Artery		Intra-parenchymal Artery	
		PSV	RI	PSV	RI
Sperm count	r	0.080	0.02	0.085	-0.15
	p	0.54	0.88	0.52	0.24
Progressive motility	r	0.050	-0.236	-0.205	-0.28
	p	0.70	0.070	0.117	0.029
Non-progressive motility	r	0.224	0.035	-0.104	-0.206
	p	0.085	0.793	0.429	0.114
Immotile	r	0.027	0.136	0.173	0.199
	p	0.81	0.299	0.186	0.128
Morphology	r	0.185	-0.061	-0.14	-0.156
	p	0.156	0.644	0.287	0.234

p, PSV, and RI stand for probability value, peak systolic velocity, and resistive index, respectively.

**Table 5:** Correlation coefficient (r) between semen quality parameters and diameters of testes and epididymis in varicocele patients (n = 60).

Parameter		Testicular Diameter		Epididymal Diameter
		Longitudinal	Transverse	Transverse
Sperm count	r	0.042	0.204	0.103
	p	0.750	0.118	0.431
Progressive motility	r	-0.094	0.077	0.031
	p	0.473	0.560	0.814
Non-progressive motility	r	-0.020	0.161	0.076
	p	0.880	0.219	0.562
Immotile	r	-0.001	-0.106	-0.017
	p	0.994	0.420	0.896
Morphology	r	0.031	0.032	0.010
	p	0.813	0.807	0.937

p stands for probability value.

## Data Availability

The data used to support the findings of this study are included within the article.
